# Supplementary material for: A Comparison of Rice Root Microbial Dynamics in Organic and Conventional Paddy Fields
Source: Microorganisms. 2024 Dec 29;13(1):41. doi: 10.3390/microorganisms13010041 (PMC11768080; doi:10.3390/microorganisms13010041)
Supplement: Supplementary file 1 [file microorganisms-13-00041-s001.zip › Fig.S2.pdf]

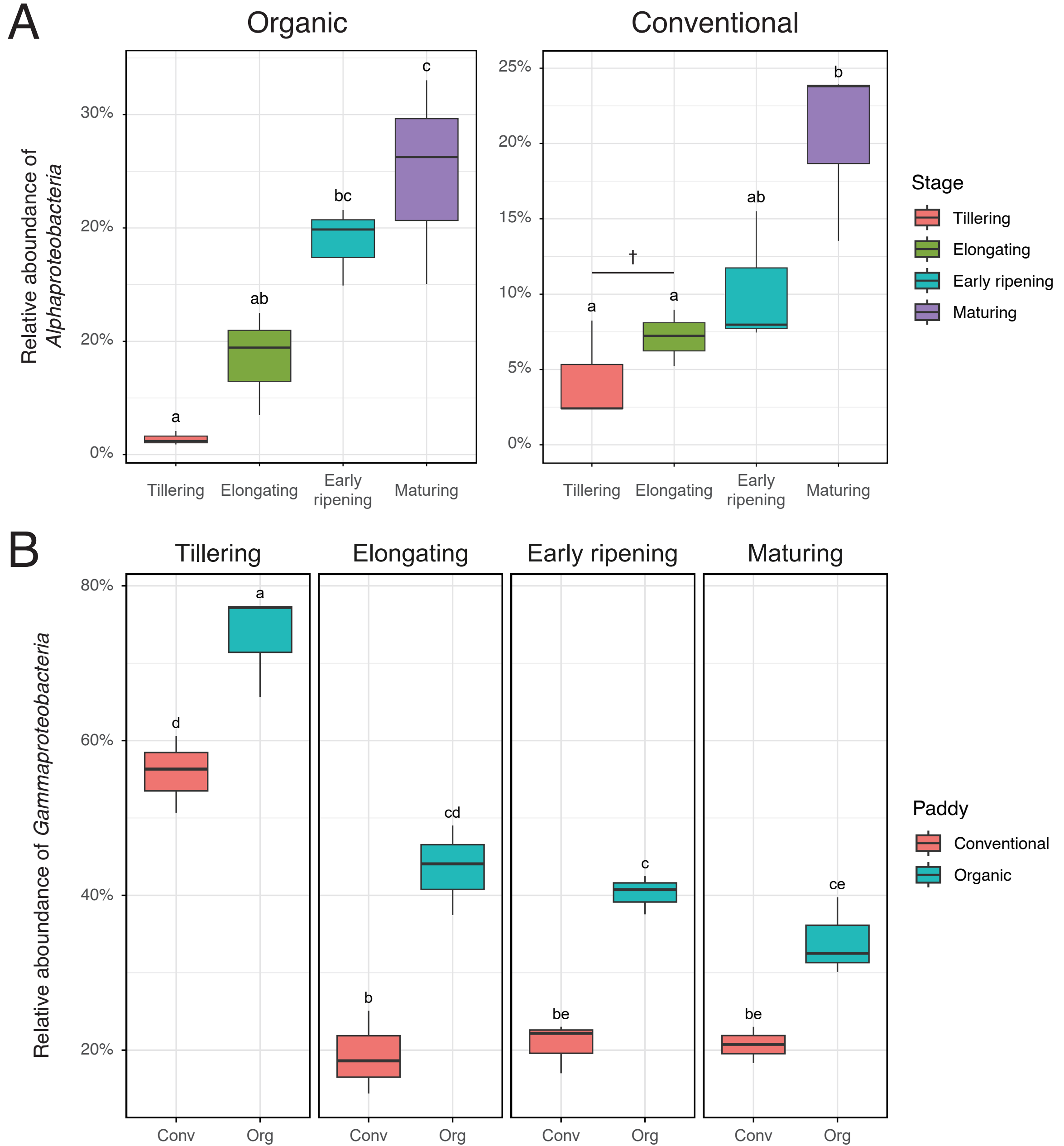

A
Organic
Conventional
Relative aboundance of Alphaproteobacteria
30%
20%
20%
0%
25%
20%
15%
10%
5%
0%
a
ab
bc
c
a
a
ab
b
†
Tillering
Elongating
Early ripening
Maturing
Stage
Tillering
Elongating
Early ripening
Maturing
B
Tillering
Elongating
Early ripening
Maturing
Relative aboundance of Gammaproteobacteria
80%
60%
40%
20%
d
a
b
cd
be
c
be
ce
Conv
Org
Paddy
Conventional
Organic
