# Supplementary material for: A Comparison of Rice Root Microbial Dynamics in Organic and Conventional Paddy Fields
Source: Microorganisms. 2024 Dec 29;13(1):41. doi: 10.3390/microorganisms13010041 (PMC11768080; doi:10.3390/microorganisms13010041)
Supplement: Supplementary file 1 [file microorganisms-13-00041-s001.zip › Table S1-5.pdf]

Table S1 Soil physicochemical property analysis methods

Parameter	Unit	Method
pH	-	Glass electrode method<br>(1:5 w/w with deionized water)
Electrical Conductivity (EC)	mS/cm	Electrical conductivity meter<br>(1:5 w/w with deionized water)
Bulk Density	-	Gravimetric method
Soil Texture	-	Simplified judgment method
Total Nitrogen (TN)	%	Macro analyzer (JM 1000 CN)
Total Carbon (TC)		Macro analyzer (JM 1000 CN)
Ammonium Nitrogen (NH_4-N)	mg/100g	Indophenol method
Nitrate Nitrogen (NO_3-N)		Alkaline reduction-diazotization method
Inorganic Nitrogen (IN)		Sum of NH_4-N and NO_3-N
Avaliable Nitrogen (AN)		Kjeldahl method
Available Phosphorus (P_2O_5)		Murphy-Riley colorimetric method
Exchangeable Potassium (K_2O)		Flame photometry
Exchangeable Calcium (CaO)		Ortho-cresolphthalein complexone method
Exchangeable Magnesium (MgO)		Xylidyl Blue I (XB-1) method
Available Silica (SiO_2)		Molybdenum blue method
Phosphate Absorption Coefficient		Vanadomolybdate method
Cation Exchange Capacity (CEC)	meq/100g	Indophenol method
Free Iron Oxide (Fe_2O_3)	%	o-Phenanthroline method
Soil Organic Matter (SOM)		Kumada modification method
Potassium Saturation Percentage		Atomic absorption spectroscopy (AAS)
Lime Saturation Percentage		Atomic absorption spectroscopy (AAS)
Magnesium Saturation Percentage		Atomic absorption spectroscopy (AAS)
Base Saturation Percentage		Atomic absorption spectroscopy (AAS)
Ca/Mg Ratio	-	-
Mg/K Ratio	-	-
Soluble Boron (B)	mg/kg	Azomethine-H method
Exchangeable Manganese (Mn)		Atomic absorption spectroscopy (AAS)
Exchangeable Zinc (Zn)		Atomic absorption spectroscopy (AAS)
Soluble Copper (Cu)		Atomic absorption spectroscopy (AAS)

Table S2. Results of three-way ANOVA examining the effects of compartment, paddy type, and developmental stage on bacterial Shannon index.

Source	Sums-Sq	df	Mean-Sq	F	p
**Main Effects**					
Compartment[1]	119.26	1	119.26	453.801	***
Paddy[2]	1.19	3	1.19	4.545	*
Stage[3]	28.98	1	9.66	36.762	***
**2-way Interactions**					
Compartment*Paddy	1.43	1	1.43	5.425	*
Compartment*Stage	10.67	3	3.56	13.538	***
Paddy*Stage	4.72	3	1.57	5.990	**
**3-way Interactions**					
Compartment*Paddy*Stage	5.62	3	1.87	7.124	***

Significant results are marked with asterisks: * indicates $p < 0.05$, ** indicates $p < 0.01$, and *** indicates $p < 0.001$.

[1]Rice root endosphere, Bulk soil;

[2]Organic, Conventional;

[3]Tillering, Elongating, Early ripening, Maturing.

Table S3. **Results of three-way ANOVA examining the effects of compartment, paddy type, and developmental stage on bacterial Observed features index.**

Source	Sums-Sq	df	Mean-Sq	F	p
**Main Effects**					
Compartment[1]	5208736	1	5208736	336.001	***
Paddy[2]	3104	1	3104	0.200	ns
Stage[3]	625373	3	208458	13.447	***
**2-way Interactions**					
Compartment*Paddy	16725	1	16725	1.079	ns
Compartment*Stage	288689	3	96230	6.207	**
Paddy*Stage	15162	3	15054	0.326	ns
**3-way Interactions**					
Compartment*Paddy*Stage	45710	3	15237	0.983	ns

Significant results are marked with asterisks: ** indicates $p < 0.01$, *** indicates $p < 0.001$, and "ns" indicates no significant difference.

[1]Rice root endosphere, Bulk soil;

[2]Organic, Conventional;

[3]Tillering, Elongating, Early ripening, Maturing.

Table S4. **Results of three-way ANOVA examining the effects of compartment, paddy type, and developmental stage on fungal Shannon index.**

Source	Sums-Sq	df	Mean-Sq	F	p
**Main Effects**					
Compartment[1]	116.5	1	116.5	214.724	***
Paddy[2]	2.76	1	2.76	5.081	*
Stage[3]	3.09	3	1.03	1.899	ns
**2-way Interactions**					
Compartment*Paddy	1.28	1	1.28	2.354	ns
Compartment*Stage	1.44	3	0.48	0.887	ns
Paddy*Stage	2.36	3	0.79	1.45	ns
**3-way Interactions**					
Compartment*Paddy*Stage	2.18	3	0.73	1.340	ns

Significant results are marked with asterisks: * indicates $p < 0.05$, *** indicates $p < 0.001$, and "ns" indicates no significant difference.

[1]Rice root endosphere, Bulk soil;

[2]Organic, Conventional;

[3]Tillering, Elongating, Early ripening, Maturing.

Table S5. Results of three-way ANOVA examining the effects of compartment, paddy type, and developmental stage on fungal Observed features index.

Source	Sums-Sq	df	Mean-Sq	F	p
**Main Effects**					
Compartment[1]	119102	1	119102	422.346	***
Paddy[2]	1485	1	1485	5.267	*
Stage[3]	2383	3	794	2.817	ns
**2-way Interactions**					
Compartment*Paddy	111	1	111	0.394	ns
Compartment*Stage	630	3	210	0.745	ns
Paddy*Stage	678	3	226	0.801	ns
**3-way Interactions**					
Compartment*Paddy*Stage	1890	3	630	2.234	ns

Significant results are marked with asterisks: * indicates $p < 0.05$, *** indicates $p < 0.001$, and "ns" indicates no significant difference.

[1]Rice root endosphere, Bulk soil;

[2]Organic, Conventional;

[3]Tillering, Elongating, Early ripening, Maturing.
