# Supplementary figures and images for: A Comparison of Rice Root Microbial Dynamics in Organic and Conventional Paddy Fields
Source: Microorganisms. 2024 Dec 29;13(1):41. doi: 10.3390/microorganisms13010041 (PMC11768080; doi:10.3390/microorganisms13010041)

A

## Bacterial genus number

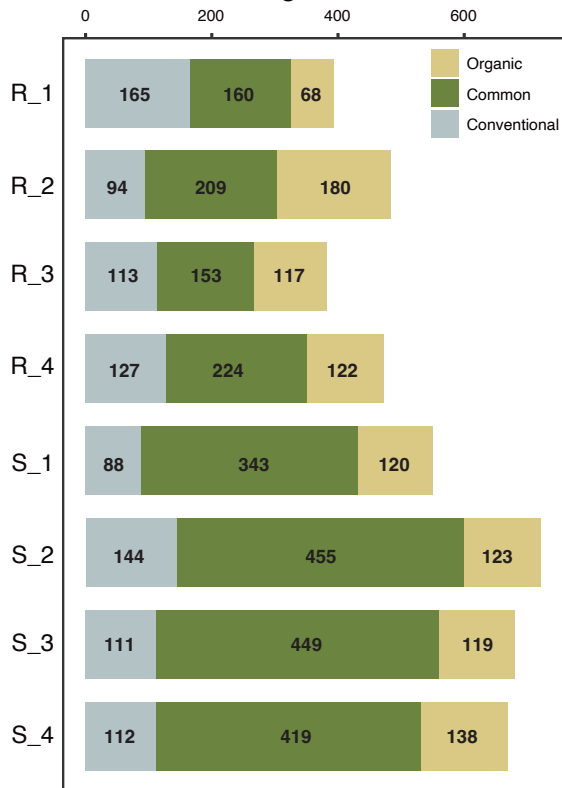

B

## Fungal genus number

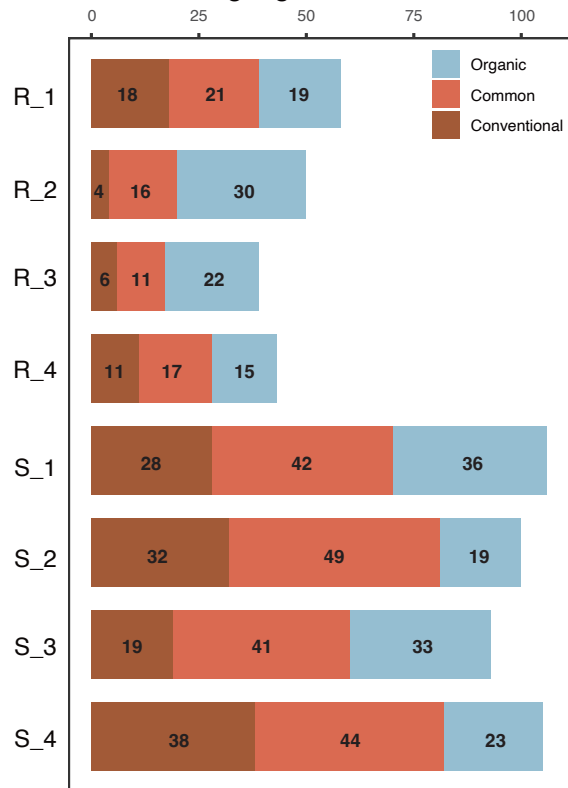

Supplement: Supplementary file 1 [file microorganisms-13-00041-s001.zip › Fig.S1.pdf]

A

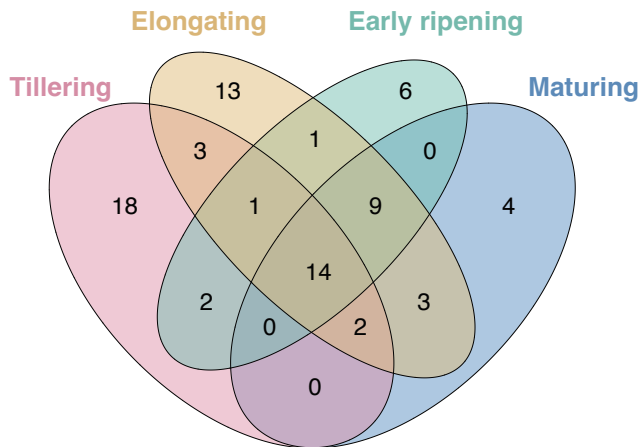

B

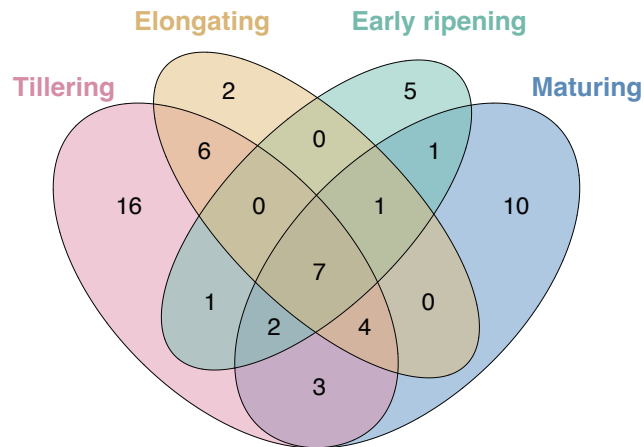

C

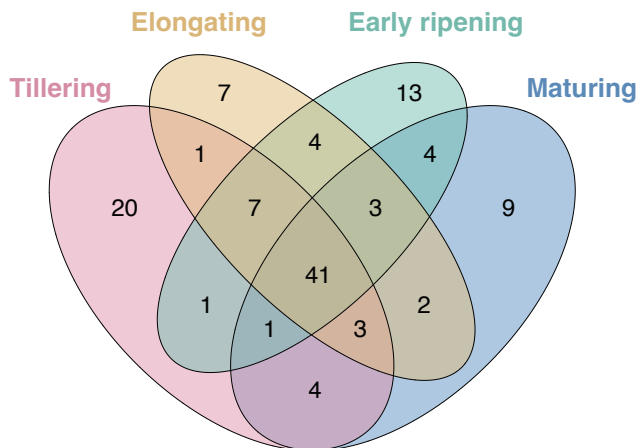

D

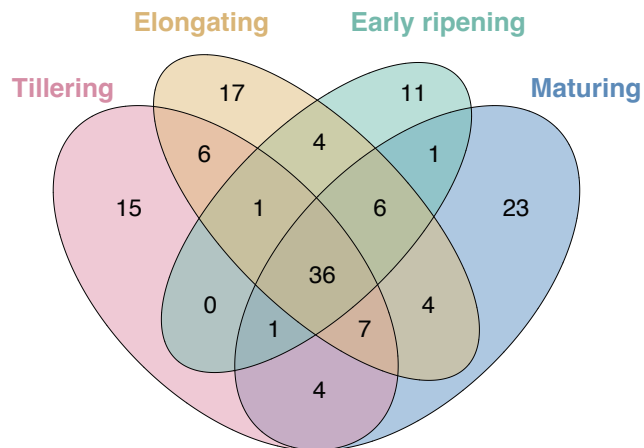

Supplement: Supplementary file 1 [file microorganisms-13-00041-s001.zip › Fig.S3.pdf]

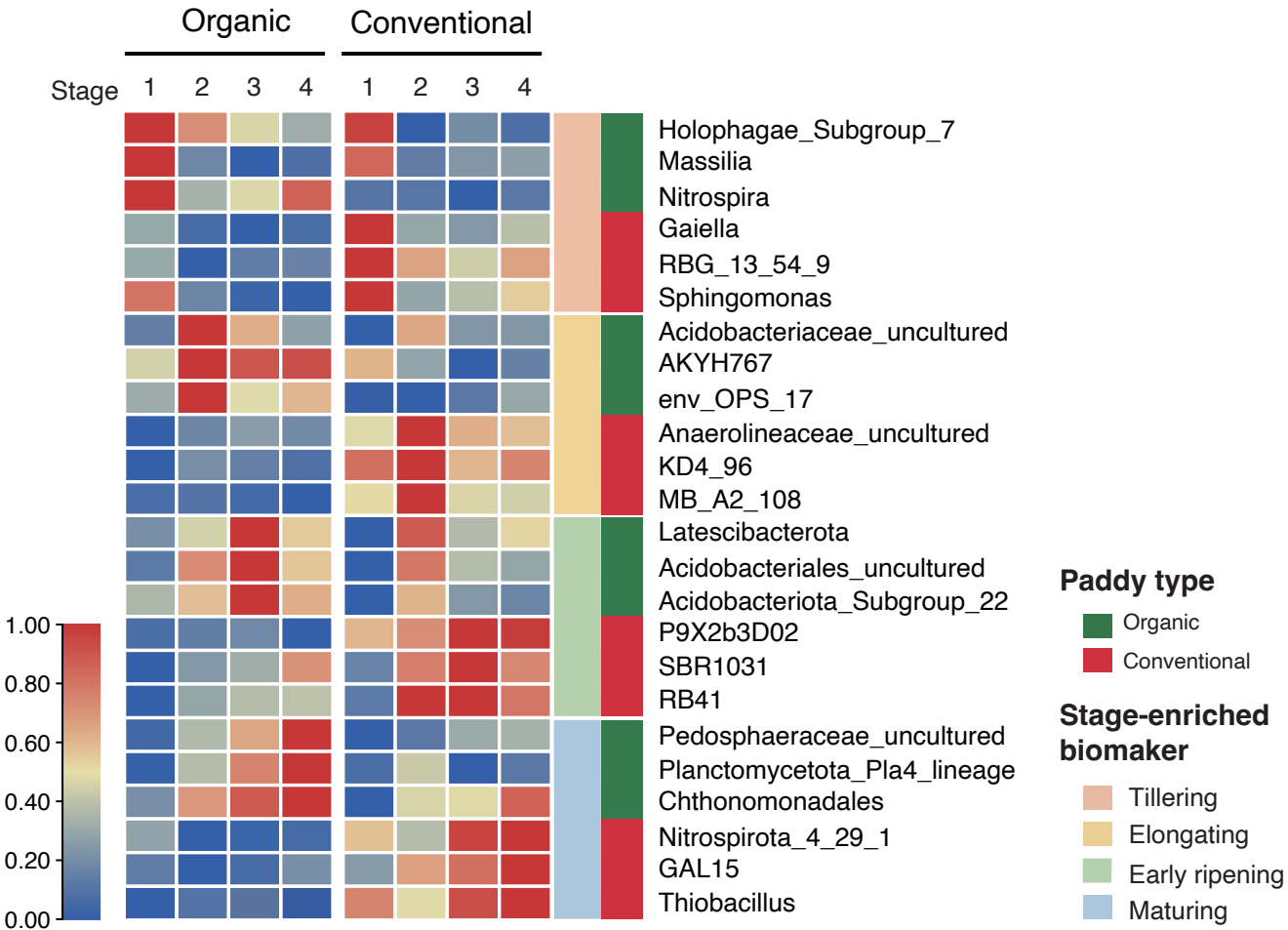

Supplement: Supplementary file 1 [file microorganisms-13-00041-s001.zip › Fig.S4.pdf]

A

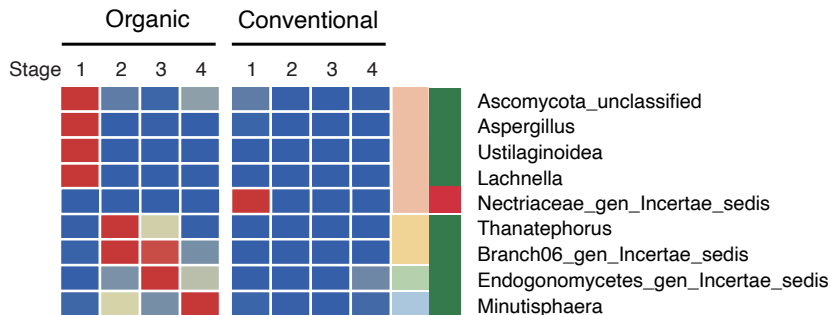

B

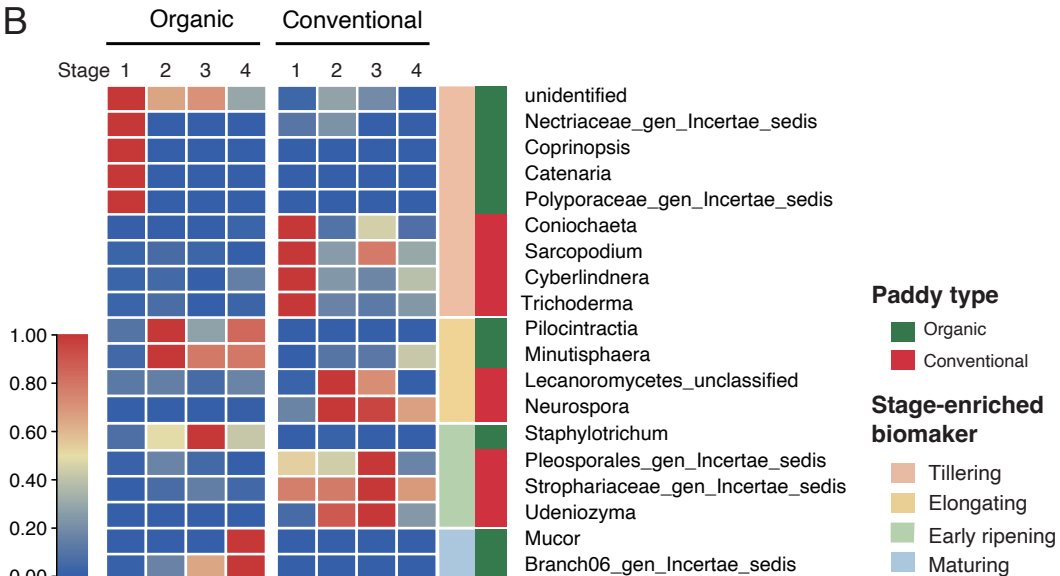

Supplement: Supplementary file 1 [file microorganisms-13-00041-s001.zip › Fig.S5.pdf]

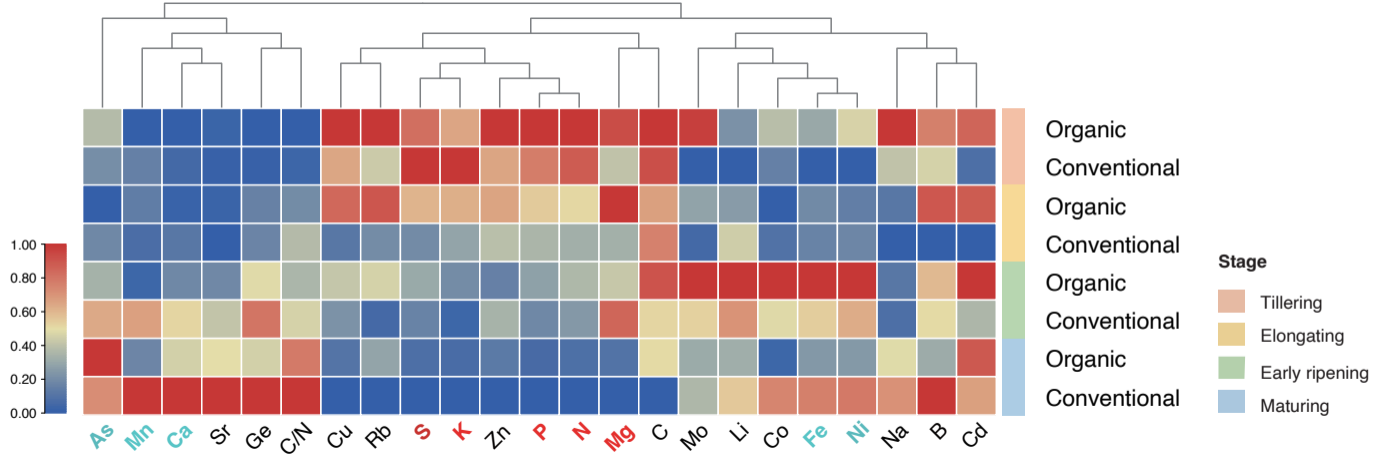

Supplement: Supplementary file 1 [file microorganisms-13-00041-s001.zip › Fig.S6.pdf]

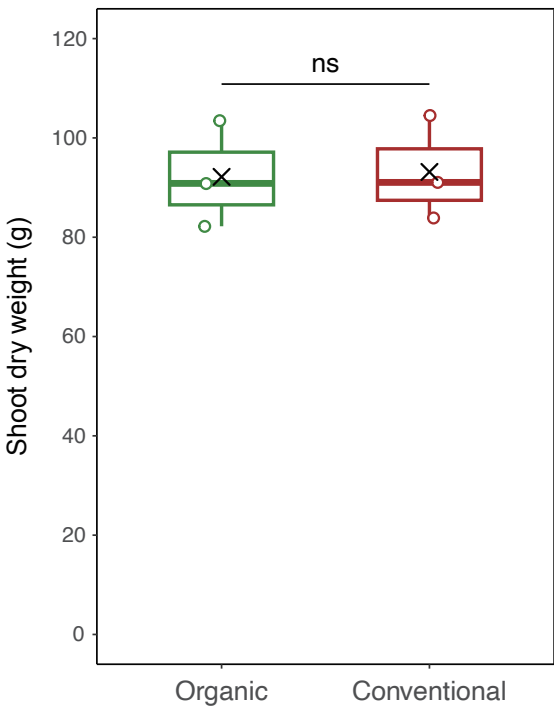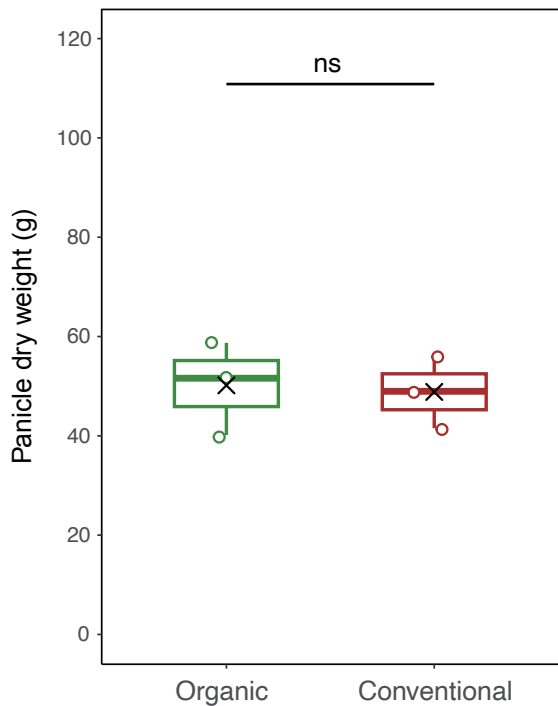

Supplement: Supplementary file 1 [file microorganisms-13-00041-s001.zip › Fig.S7.pdf]
